# The nutritional content and cost of supermarket ready-meals. Cross-sectional analysis^☆^

**DOI:** 10.1016/j.appet.2015.04.069

**Published:** 2015-09-01

**Authors:** Jennifer Remnant, Jean Adams

**Affiliations:** Institute of Health & Society, Newcastle University, Baddiley-Clark Building, Richardson Road, Newcastle upon Tyne NE2 4HH, UK

**Keywords:** Diet, Nutrition, Food, Economic cost

## Abstract

*Background:* Over-reliance on convenience foods, including ready-meals, has been suggested as one contributor to obesity. Little research has systematically explored the nutritional content of supermarket ready-meals. We described the nutritional content and cost of UK supermarket ready-meals. *Methods:* We conducted a survey of supermarket own-brand chilled and frozen ready-meals available in branches of ten national supermarket chains in one city in northern England. Data on price, weight and nutritional content of meals in four ranges (‘healthier’, luxury, economy and standard) and of six types (macaroni cheese, meat lasagne, cottage pie, chicken tikka masala, fish pie, and sweet and sour chicken) were collected. Nutritional content was compared to ranges used to identify low, medium and high fat, saturated fat, sugar and salt in nationally recommended front-of-pack labelling. *Results:* 166 ready-meals were included from 41 stores. Overall, ready-meals were high in saturated fat and salt, and low in sugar. One-fifth of meals were low in fat, saturated fat, salt and sugar, including two-thirds of ‘healthier’ meals. Meals that were low for three out of the four front-of-pack nutrients were the cheapest. *Conclusions:* Supermarket ready-meals do not have a healthful nutritional profile overall. However, a number of healthier meals were available – particularly amongst meals specifically marked as ‘healthier’. There was little evidence that healthier meals necessarily cost more. Further effort is required to encourage producers to improve the nutritional profile of the full range of ready-meals, and not just those specifically labelled as ‘healthier’.

## Highlights

• Over-reliance on convenience foods, including ready-meals, may contribute to obesity.
• We surveyed supermarket own-brand ready-meals in ten UK supermarkets.
• Overall, ready-meals tended to be high in saturated fat and salt, and low in sugar.
• 20% of meals were low in fat, saturated fat, salt and sugar.
• There was little evidence that healthier meals necessarily cost more.

## Introduction

The endemic nature of obesity in many countries (Ng et al., 2014) has led to increasing attention being paid to dietary practices and choices. One growing area of concern is a perceived decline in home cooking with an increasing reliance on convenience foods, including ready-meals (Lichtenstein & Ludwig, 2010).

Ready-meals have been defined as pre-prepared main courses that can be reheated in their container, requiring no further ingredients, and needing only minimal preparation before consumption. The UK has one of the most dynamic ready-meal markets, accounting for more than £1.4 bn in annual sales in the year to January 2014 – a 1.5% year-on-year increase (Chilled Food Association, 2014). UK data suggest that in 2003 almost two-thirds of UK households consumed some ready-meals (Reed, McIlveen-Farley, & Strugnell, 2003); and in 2006 40% of households ate ready-meals at least once per week (Mahon, Cowan, & McCarthy, 2006). More recent, detailed and population-representative data on frequency of consumption are not available.

More than 90% of ready-meals sold in the UK are supermarket own-brand products (Key Note, 2013). Most supermarkets ‘brand’ their own-brand products into premium or luxury, ‘healthier’, economy or value, as well as standard ranges (Celnik, Gillespie, & Lean, 2012). Fresh and frozen varieties of many meals are available across these ranges.

Consumers' reasons for choosing ready-meals particularly focus on the perceived convenience and value for money compared to home cooking (Ahlgren, Gustafsson, & Hall, 2005; Costa, Schoolmeester, Dekker, & Jongen, 2007; de Boer, McCarthy, Cowan, & Ryan, 2004; Mahon et al., 2006; Mia, Inga-Britt, & Gunnar, 2006). However, the health benefits of ‘healthy’ ranges and any nutritional benefit or loss associated with the price differentials of premium and economy ranges are not clear.

Consumption of ready-meals has been associated with higher body weight (van der Horst, Brunner, & Siegrist, 2011). This is likely because ready-meals tend to contain high levels of fat and saturated fat (Celnik et al., 2012; de Boer et al., 2004). Although a number of previous studies of the nutritional content of supermarket ready-meals have been conducted, these have all been limited in scope (Anderson, Wrieden, Tasker, & Gregor, 2008; Celnik et al., 2012; Howard, Adams, & White, 2012). No previous study has systematically explored the nutritional content of the full range of popular ready-meals. Nor has any study explored the cost of ready-meals and any relationship between cost and nutritional content. Thus, our aim was to describe the nutritional content of supermarket ready-meals and explore associations between cost and nutritional content.

## Methods

We conducted a survey of the price and nutritional content of supermarket own-brand ready-meals sold in branches of large supermarket chains in one city in Northern England (current population about 280,000).

### Selection of supermarket chains

Outlets operated by ten supermarkets were included in the study: *Aldi*, *Asda*, *Cooperative Food*, *Iceland*, *Lidl*, *Marks & Spencer*, *Morrisons*, *Sainsbury*'s, *Tesco*, *Waitrose*. Together, these accounted for a combined grocery market share, at the time of data collection, of more than 95% (Statista (Kantar Worldpanel), 2014).

### Selection of ready-meals

As previously, ready-meals were defined as pre-prepared meals, supplied in the container used for cooking, with no further ingredients or preparation required other than heating (Howard et al., 2012). We restricted the sample to supermarket own-brand ready-meals intended as single servings. In all cases, both frozen and chilled ranges were searched for and included if present. One supermarket (*Lidl*) did not sell own-brand ready-meals at the time of data collection and was excluded from further consideration.

Meals in four ‘ranges’ were included – luxury, standard, value and ‘healthier’. Although the specific name of each range varied between supermarkets, it was not difficult to place all meals found in one of the four ranges based on explicit branding on packages. Examples of written branding used on packages in different ranges are given in Table 1, although other aspects of branding (e.g. colour and pictures) also plays an important role in identifying meal ranges. However, not all supermarkets sold meals in all ranges and some supermarkets had more than one label within a particular range. In these cases all eligible meals were included.

We included six meal types: macaroni cheese, meat lasagne, cottage pie, fish pie, chicken tikka masala, and sweet and sour chicken. These reflect four meal types (macaroni cheese, meat lasagne, cottage pie, and chicken tikka masala) included in previous work as ‘popular choices’ (Celnik et al., 2012), as well as two additional meal types (fish pie and sweet and sour chicken) that reflect expanding tastes in UK ready-meal consumption. Brief descriptions of each meal type are provided in Table 1.

Within each supermarket, we identified the number of eligible ranges present and assumed that all six eligible meal types were available in both chilled and frozen versions within these ranges. This gave a total number of potentially eligible meals. However, we cannot be sure that all these potentially eligible meals were produced and sold. The number of eligible meals found in all stores visited, in comparison to the total number of potentially eligible meals, is described in Table 1. At least one representative of each meal type was present in each meal range, and vice versa.

### Data collection

All branches of included supermarkets within the study city boundaries were identified from supermarket websites and visited by one researcher over one week in April 2013. In each store, the researcher identified all ready-meals that met the inclusion criteria and recorded the price, weight and nutritional information shown on packaging. Specifically, total energy, fat, saturated fat, carbohydrate, sugar, protein, fibre and salt were recorded. Nutrient content per 100 g of product was also recorded.

When meals that had previously been encountered during data collection were found again in a subsequent branch, weight and nutritional information were not re-recorded. Price was recorded on all occasions to allow for the potential for ‘price flexing’ – variations in price of the same product across different branches of the same chain. In these cases the average price of the meal across all branches in which it was found was calculated for use in analysis.

A second researcher visited a 10% (n = 4) random sample of included stores during the same week as the first researcher and collected data independently. There was 100% agreement between researchers in the meals identified for inclusion and the price, weight and nutritional content of included meals.

### Analysis

All analyses were conducted at the meal level. As there was evidence that some variables were not normally distributed, non-parametric methods were used throughout.

The cost, weight and nutritional content of meals overall and within meal ranges and types were described using median and interquartile ranges. Differences between meal ranges and types were explored using Kruskall–Wallis tests. As not all ready-meals have the same weight, similar analyses were conducted for both total nutritional content and nutritional content per 100 g.

Median nutritional content per 100 g was compared to current UK guidance on front-of-pack nutrition, or ‘traffic light’, labelling – this indicates ranges for red/high, amber/medium and green/low content of fat, saturated fat, sugar and salt (Department of Health, Foods Standards Agency, Welsh Government, & The Scottish Government, 2013). The number of meals rated as ‘low’ for one, two, three or all four of these nutrients was also calculated. Differences in the number of nutrients that meals were rated ‘low’ for across meal ranges and types were explored using chi-squared tests.

Associations between price and both weight and nutritional content, overall and within meal ranges and types, were explored using Spearman rank correlation tests.

All analyses were conducted in Stata v13.0. As a large number of statistical tests were performed, a p-value of <0.01 was taken to indicate statistical significance.

### Research ethics

Ethical permission was not required for this study as it did not include any human or animal participants.

## Results

Forty one supermarkets met the inclusion criteria and were visited. Out of 360 potentially eligible meals, 166 (46%) were found and included in the analysis (see Table 1). There was no difference in the proportion of potentially eligible meals available by meal type, but there was evidence that availability varied by meal range. Meals in the standard range were most likely, and meals in the luxury range least likely, to be available.

Table 2 summarises the total cost, weight and nutritional content of included meals and how these varied by meal range and type. Overall, meals cost a median of £2.20 ($US3.52; €2.80) and contained a median of 450 kcal. All variables, except sugar and fibre content, varied significantly across meal ranges. Cost was highest in luxury ranges and lowest in value ranges. However, value ranges also tended to be slightly lighter than other ranges. There was evidence that meals in ‘healthier’ ranges contained less total energy, fat, saturated fat and salt that meals in other ranges – indicating that they were ‘healthier’ on a number of parameters. However, fibre did not vary between meal ranges. In contrast, meals in the luxury ranges tended to have the least healthy profiles with the highest total energy, fat, saturated fat and salt content. Meals in value ranges were particularly low in protein.

Although cost, weight and salt content did not vary significantly between meal types, all other aspects of nutritional content did. Total energy was lowest in fish pie and cottage pie and highest in macaroni cheese. Fat and saturated fat were lowest in sweet and sour chicken and highest in macaroni cheese. Sugar was lowest in fish pie and cottage pie, but highest in sweet and sour chicken. Protein was highest in chicken tikka masala and lowest in cottage pie. Fibre was highest in chicken tikka masala and cottage pie, but lowest in macaroni cheese.

To take account of differences in product weight, Table 3 summarises the relative nutritional content per 100 g of product of included meals. In general, and despite variations in weight, differences across groups in Table 3 reflected those in Table 2.

Shading in Table 3 reflects current UK guidance on front-of-pack nutrition labelling (Department of Health et al., 2013). Overall, meals were rated as medium for fat, high for saturated fat and salt, and low for sugar. As a group, meals in the ‘healthier’ ranges were low for fat, saturated fat and sugar, and medium for salt. Meals in luxury ranges were high for fat, saturated fat and salt, and low in sugar. Macaroni cheese meals had the least healthy profile being rated, as a group, as high in fat, saturated fat and salt, but low in sugar. In contrast, sweet and sour chicken meals were low in fat and saturated fat but high in sugar and salt.

Table 4 shows the number of meals in each category that were rated as low for one, two, three or all four of the front-of-pack nutrients. Overall, one-fifth of meals were low for all four nutrients. All meals were rated as low for at least one front-of-pack nutrient. The only significant differences in the number of front-of-pack nutrients that meals were low for were by meal range. No meals in the ‘luxury’ ranges were rated as low for all four nutrients and two-thirds were only low for one nutrient. In contrast, no meals in the ‘healthier’ ranges were low for just one nutrient and two-thirds were low for all four.

Correlations between price and both weight and nutritional content are shown in Table 5. Overall there was a strong positive correlation between price and protein content; moderate positive correlations between price and weight, energy and fat content; and weak positive correlations between price and saturated fat and fibre content. Similar correlations were seen within meal ranges and types.

Meals that were low for one front-of-pack nutrient cost a median of £2.35 (inter-quartile range £1.71–£3.48), those that were low for two cost £2.20 (£1.00–£2.80), those that were low for three cost £1.25 (£1.00–£2.20) and those that were low for all four cost a median of £2.20 (£1.00–£2.50; χ^2^ = 15.26, p < 0.002).

## Discussion

### Summary of findings

This is the first study we are aware of to systematically explore the nutritional content and cost of the full landscape of supermarket own-brand ready-meals. Across 41 branches of nine national supermarkets, we found 166 ready-meals that met our inclusion criteria. Nutritional content varied substantially according to meal range and type. Overall, meals were categorised as high in saturated fat and salt, and low in sugar according to current UK guidance for front-of-pack nutritional labelling (Ahlgren et al., 2005). One-fifth of all meals were rated as low for all four front-of-pack nutrients, including two-thirds of meals in ranges specifically marketed as ‘healthier’, but none of the meals specifically marketed as ‘luxury’. The cost of meals was positively associated with weight, total energy, fat, saturated fat, protein and fibre. Meals that were rated as low for three out of the four front-of-pack nutrients were the cheapest, and those that met only one the most expensive.

### Strengths and limitations of methods

Our methods represent a significant improvement on previous methods used to study the nutritional content of supermarket ready-meals. Unlike previous work (Anderson et al., 2008; Celnik et al., 2012; Howard et al., 2012), we included a much fuller range of supermarket ready-meals currently available in the UK, identified using systematic methods, and provided a detailed analysis of nutritional content. In particular, we included a wider range of supermarkets, meal ranges, and meal types than previously (Anderson et al., 2008; Celnik et al., 2012; Howard et al., 2012). Uniquely we also explored the cost of meals, and associations between cost and nutritional content.

The range of nutrients included does not include some important micro-nutrients, or other aspects of diet. In particular, we did not have information on the fruit and vegetable content of included ready-meals.

This study was conducted in one city in Northern England. Whilst the nutritional content of supermarket ready-meals stated on packaging is unlikely to vary across the UK, there may be small variations in actual nutritional content from batch to batch, and between meals produced in different locations. We relied entirely on the nutritional content as stated on packaging and did not independently verify that this was accurate. In the UK, nutritional information on food packaging is permitted by law to vary by 20% from the values, to allow for fluctuation in manufacturing processes (Cantelo, 2010). It is, therefore, possible that there may be some error in the nutritional information we used.

In order to capture variations in price across different branches of the same supermarket, we collected price data from all branches of included supermarket chains in the study city. This is likely to increase the generalisability of price data. It is also possible that ready-meal availability varies across the country and that studies in different cities would have identified different eligible meals. The 100% inter-rater agreement on all variables indicates that our data are likely to be highly reliable.

Manufacturers of processed foods are constantly reformulating products (Consensus Action on Salt & Health, 2007). Similarly the price of foods is not constant. We collected data over a period of only one week in order to avoid time-related changes in price or nutritional content. However, it is possible that both the price and nutritional content of supermarket own-brand ready-meals have changed in the time since data collection.

We did not have any information on sales and were not able to take into account how popular different ready-meals in the sample were. Nor were we able to draw any conclusions on how the ready-meals in the sample contribute to the total diet of consumers: it is not necessarily the case that ready-meals are the least nutritious component of consumers' diets.

### Interpretation and implications of findings

Previous work has attempted to compare the absolute nutritional content of ready-meals with nutritional standards for meals (Anderson et al., 2008; Celnik et al., 2012). One important finding was that ready-meals often contain substantially fewer calories than is recommended for a meal. Whilst we considered conducting a similar analysis, ready-meals are probably more sensibly considered ‘ready-main-courses’, than complete meals. This may explain why the total energy content is less than might be expected. As such, we chose to use the cut-offs for front-of-pack nutritional labelling (based on nutritional content per 100 g) instead of whole meal nutrient standards (Crawley, 2005). These also allow for comparisons between products of different sizes.

As in previous work, we found that ready-meals tended to be high in fat, saturated fat and salt (Howard et al., 2012). Unlike eating take-away meals at home, which are recognised by consumers to be less healthy, occasional ‘treats’ (Wrigley, Warm, Margetts, & Lowe, 2004), ready-meals are primary seen as a convenient alternative to home cooking (Ahlgren et al., 2005; Costa et al., 2007; de Boer et al., 2004; Mahon et al., 2006; Mia et al., 2006). Recent population-representative data from the UK suggests that around one-fifth of adults consume take-away meals at home once per week or more often (Adams et al., 2015). Although such high quality data on the frequency of consumption of ready-meals is not available, in 2006 it was estimated that 40% of UK households ate such meals at least once per week (Mahon et al., 2006). The population impact and public health implications of ready-meals may, therefore, be much larger than those of take-aways – despite the latter receiving much more research and media attention. Further work exploring the relative contribution of different foods prepared outside the home, but consumed inside the home, to total diet will help guide intervention developers to the area most likely to achieve the largest population impact.

We found that the nutritional content of ready-meals varied across meal range and type. In particular, we found that meals specifically labelled as ‘healthier’ were rated as low in fat, saturated fat and sugar and medium in salt overall, and were much more likely to achieve ‘low’ ratings of all four front-of-pack nutrients than meals in any other category. This suggests that healthier alternatives are available within the ready-meal sector and a simple public health message to avoid all ready-meals may be inappropriate. However, it is worth noting that meals in the ‘healthier’ ranges were not necessarily always ‘low’ in all four front-of-pack nutrients – although consumers may, perhaps, expect this. Further research is required to determine whether consumers are being misled by current branding and whether stricter rules are required on what circumstances ‘healthier’ branding can be used by food manufacturers.

Qualitative research has found that being seen to eat healthily and eating foods overtly branded as ‘healthy’ can be socially damaging and is associated with being less popular in both young people and adults – particularly those from less affluent backgrounds (O'Neill, Rebane, & Lester, 2004; Stead, McDermott, MacKintosh, & Adamson, 2011). Further work is required to understand how to increase consumer acceptance of healthier products. Avoiding overtly branding such products as ‘healthier’ could also be productive.

It is difficult to untangle the relationship between consumer food preferences and manufactured food availability. It is possible that preferences are driven by what is available, or manufacturers may make available what is preferred. In practice, a combination of both of these two scenarios is likely to be operating. This suggests that changing the ‘food supply’ towards healthier manufactured food will not necessarily lead to changes in what consumers eat. It is possible that if the content of products is changed to be healthier, consumers will change what products they choose. The association of widespread reductions in the salt content in manufactured food across the UK with reductions in overall salt intake suggests that it is possible for healthy changes in ‘food supply’ to impact on population diets (He, Brinsden, & MacGregor, 2014).

The finding that healthier ready-meals are available reinforces the sophistication of the food industry in terms of product formulation. Previous studies have reported mixed findings in terms of reduction in salt content of ready-meals over time, suggesting that consistent progress is not taking place (Christoforou, Dunford, & Neal, 2013; Howard et al., 2012). Given that it is clearly possible to produce healthier ready-meals, more pressure could be placed on the ready-meal industry to improve the nutritional profile of all meals, and not just those specifically labelled as ‘healthier’. As the cost of meals in the ‘healthier’ ranges was not substantially greater than those in ‘standard’ ranges, improving the nutritional profile of ready-meals would seem to be unlikely to lead to any increase in cost to the consumer.

We found that the cost of ready-meals was positively associated with weight, energy, fat, saturated fat, protein and fibre. Whilst fat and saturated fat are nutrients that, in population terms, we should be consuming less of, fibre is a nutrient that we should be consuming more of. Thus, consumers who choose more expensive ready-meals are, in general, receiving a mixed health benefit for this expense. This is reinforced by the finding that, in terms of nutrients included in front-of-pack labelling, the cheapest meals were those rated as low on three out of four of these nutrients.

### Conclusions

Supermarket ready-meals tend to be high in saturated fat and salt, medium in total fat, and low in sugar according to current UK guidance for front-of-pack nutritional labelling (Department of Health et al., 2013). However, nutritional content varied substantially and a number of meals that were low in all these nutrients were available, particularly amongst meals specifically marked as ‘healthier’. The cost of meals was positively associated with weight, energy, fat, saturated fat, protein and fibre, suggesting that consumers do not necessarily have to pay more for healthier meals. Further effort is required to encourage producers to improve the nutritional profile of the full range of ready-meals, and not just those specifically labelled as ‘healthier’.

## Figures and Tables

**Table 1:** Availability of supermarket ready-meals in study city.

Variable	Level (examples of written branding/description)	Labels available, n	Meals potentially eligible, n	Meals available, n (% of eligible)
Meal range	Luxury (e.g. ‘finest’, ‘extra special’, ‘taste the difference’)	14	84	23 (26)
	Standard (e.g. ‘original’, ‘classic’)	18	108	72 (67)
	Value (e.g. ‘saver’, ‘smart price’, ‘basic’)	12	72	36 (50)
	Healthy (e.g. ‘light choices’, ‘be good to yourself’, ‘good life’)	16	96	35 (36)
	χ^2^ (p-value)	–	–	12.53 (0.006)
Meal type	Chicken tikka masala (roasted chicken chunks in a spicy and creamy sauce)	60	60	28 (47)
	Cottage pie (minced beef with a mashed potato crust)	60	60	34 (57)
	Fish pie (flaked white fish in a white or cheese sauce with a mashed potato crust)	60	60	26 (43)
	Lasagne (flat pasta sheets layered with minced beef and tomato sauce, and cheese sauce)	60	60	35 (58)
	Macaroni cheese (tubular pasta pieces in a cheese sauce)	60	60	21 (35)
	Sweet and sour chicken (chicken chunks deep fried in batter, in a sweet and sour sauce)	60	60	22 (37)
	χ^2^ (p-value)	–	–	4.27 (0.512)
All	–	–	360	166 (46)

**Table 2:** Median (inter-quartile range) cost, weight and total nutritional content of supermarket ready-meals.

Variable	Level	Cost (£)	Weight (g)	Energy (kcal)	Fat (g)	Sat. fat (g)	Carb. (g)	Sugar (g)	Protein (g)	Fibre (g)	Salt (g)
Meal range	Luxury	3.50 (3.30–3.99)	400 (400–400)	581 (444–696)	27.6 (18.5–37.2)	13.5 (10.0–18.0)	41.3 (36.4–53.2)	7.7 (4.0–10.8)	30.8 (27.2–37.2)	6.4 (4.8–8.1)	2.4 (2.0–2.8)
	Healthy	2.20 (1.00–2.29)	400 (374–400)	344 (302–394)	8.0 (5.2–9.6)	3.6 (2.0–4.8)	51.5 (42.0–57.2)	8.8 (6.0–10.8)	24.1 (20.3–27.7)	5.2 (4.4–6.8)	1.6 (1.4–2.0)
	Standard	2.12 (1.50–2.47)	400 (400–450)	520 (452–634)	20.8 (14.4–25.8)	9.6 (5.1–12.1)	57.8 (44.2–82.6)	8.4 (4.8–15.4)	28.2 (23.6–33.8)	5.1 (3.6–6.8)	2.3 (1.9–2.5)
	Value	0.85 (0.80–1.00)	400 (300–400)	405 (291–464)	13.7 (7.4–20.7)	7.0 (3.1–10.1)	46.6 (35.9–55.7)	5.7 (3.7–11.0)	18.2 (14.6–23.2)	4.5 (3.3–5.9)	1.9 (1.6–2.1)
	χ^2^ (p-value)	96.11 (<0.001)	33.16 (<0.001)	63.27 (<0.001)	70.28 (<0.001)	52.62 (<0.001)	16.28 (0.001)	5.55 (0.136)	53.58 (<0.001)	10.21 (0.017)	41.00 (<0.001)
Meal type	Chicken tikka masala	2.20 (1.50–3.50)	400 (400–450)	513 (409–669)	15.6 (6.6–26.5)	4.6 (2.4–8.1)	67.7 (56.9–77.6)	10.7 (8.5–13.4)	29.8 (25.9–38.3)	6.2 (4.3–9.0)	2.0 (1.6–2.7)
	Cottage pie	2.20 (1.00–2.49)	400 (400–433)	399 (300–458)	15.9 (9.2–22.1)	7.8 (4.7–11.3)	40.2 (37.0–45.7)	4.6 (2.8–6.8)	22.4 (16.0–27.9)	6.0 (4.4–7.2)	2.0 (1.6–2.3)
	Fish pie	2.20 (1.00–2.99)	400 (400–400)	393 (334–462)	15.0 (11.7–21.3)	8.7 (5.6–11.3)	40.2 (34.1–45.9)	4.3 (3.1–5.3)	25.6 (20.0–30.5)	4.4 (3.5–5.4)	2.0 (1.7–2.4)
	Lasagne	2.10 (1.00–2.39)	400 (400–400)	470 (393–609)	20.8 (12.4–28.5)	9.6 (6.6–14.0)	47.2 (39.2–51.5)	9.3 (8.0–12.4)	26.0 (23.2–31.2)	5.6 (4.4–6.6)	2.2 (1.8–2.4)
	Macaroni cheese	2.10 (1.00–2.39)	400 (360–400)	665 (449–752)	28.4 (18.7–34.8)	17.2 (9.6–20.0)	73.5 (55.7–80.5)	5.4 (4.4–7.4)	24.0 (17.2–31.0)	4.0 (3.2–5.8)	1.9 (1.6–2.1)
	Sweet and sour chicken	1.75 (1.00–3.26)	400 (400–450)	513 (437–608)	4.6 (2.2–12.6)	0.8 (0.6–1.6)	89.4 (74.4–100.7)	29.3 (23.7–34.5)	26.2 (20.0–33.6)	4.6 (3.3–7.8)	2.1 (1.6–2.5)
	χ^2^ (p-value)	5.37 (0.373)	8.06 (0.153)	42.57 (<0.001)	46.54 (<0.001)	80.43 (<0.001)	108.08 (<0.001)	113.47 (<0.001)	19.69 (0.001)	15.61 (0.008)	3.46 (0.630)
All meals	–	2.20 (1.00–2.59)	400 (400–400)	450 (388–585)	16.3 (9.2–23.5)	7.2 (3.5–11.8)	49.6 (40.5–71.8)	8.0 (4.8–12.4)	26.0 (20.4–31.2)	5.2 (3.9–6.8)	2.0 (1.6–2.4)

**Table 3:**
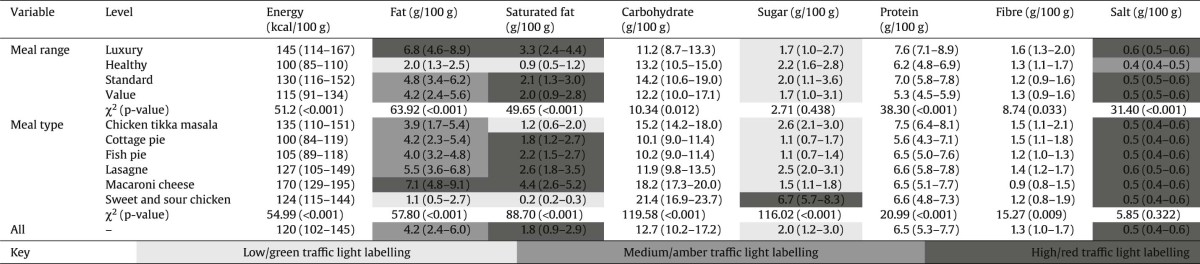
Median (inter-quartile range) nutritional content per 100 g of supermarket ready-meals.

*Note:* Shading reflects current UK guidance on front of pack nutrition, or ‘traffic light’, labelling. This is only available for fat, saturated fat, sugar and salt.

**Table 4:** Number of front-of-pack nutrients that supermarket ready-meals meet ‘low’ cut-off for.

Variable	Level	One nutrient	Two nutrients	Three nutrients	Four nutrients	Total	χ^2^ (p-value)
Meal range	Luxury, n (%)	14 (61)	8 (35)	1 (4)	0	23 (100)	–
	Healthy, n (%)	0	2 (6)	10 (29)	23 (66)	35 (100)	–
	Standard, n (%)	29 (40)	23 (32)	18 (15)	2 (3)	72 (100)	–
	Value, n (%)	5 (14)	12 (33)	11 (31)	8 (22)	36 (100)	88.73 (<0.001)
Meal type	Chicken tikka masala, n (%)	10 (36)	2 (7)	6 (21)	10 (36)	28 (100)	–
	Cottage pie, n (%)	9 (26)	9 (26)	9 (26)	7 (21)	34 (100)	–
	Fish pie, n (%)	6 (23)	6 (23)	9 (35)	5 (19)	26 (100)	–
	Lasagne, n (%)	16 (46)	9 (26)	5 (14)	5 (14)	35 (100)	–
	Macaroni cheese, n (%)	7 (33)	9 (43)	4 (19)	1 (5)	21 (100)	–
	Sweet and sour chicken, n (%)	0	10 (45)	7 (32)	5 (23)	22 (100	29.82 (0.013)
All meals	–	48 (29)	45 (27)	40 (24)	33 (20)	166 (100)	–

*Note:* Total of row percentages do not necessarily sum to 100 due to rounding error.

**Table 5:** Spearman rank correlations (p-value) between price and weight and nutritional content of supermarket ready-meals.

Variable	Level	Weight (g)	Energy (kcal)	Fat (g)	Sat. fat (g)	Carbohydrate (g)	Sugar (g)	Protein (g)	Fibre (g)	Salt (g)
Meal range	Luxury	0.14 (0.536)	0.18 (0.418)	0.29 (0.187)	0.09 (0.694)	0.17 (0.440)	−0.02 (0.942)	0.17 (0.431)	−0.10 (0.657)	0.03 (0.903)
	Healthy	0.10 (0.568)	0.22 (0.220)	0.12 (0.508)	−0.02 (0.899)	−0.12 (0.484)	0.14 (0.409)	0.39 (0.020)	0.02 (0.913)	−0.39 (0.020)
	Standard	0.24 (0.047)	0.37 (0.001)	0.20 (0.097)	0.18 (0.132)	0.05 (0.662)	0.20 (0.095)	0.56 (<0.001)	0.18 (0.122)	−0.03 (0.810)
	Value	0.48 (0.003)	0.33 (0.049)	0.14 (0.403)	0.12 (0.503)	0.24 (0.157)	0.10 (0.563)	0.54 (<0.001)	−0.10 (0.573)	0.24 (0.165)
Meal type	Chicken tikka masala	0.52 (0.005)	0.46 (0.013)	0.46 (0.013)	0.41 (0.029)	0.16 (0.420)	0.42 (0.024)	0.66 (<0.001)	0.410 (0.030)	0.31 (0.111)
	Cottage pie	0.24 (0.174)	0.40 (0.018)	0.24 (0.169)	0.29 (0.096)	0.11 (0.545)	0.37 (0.033)	0.53 (0.001)	0.49 (0.003)	0.18 (0.315)
	Fish pie	0.25 (0.213)	0.48 (0.013)	0.35 (0.075)	0.41 (0.039)	−0.29 (0.155)	0.01 (0.960)	0.52 (0.006)	0.21 (0.292)	0.26 (0.203)
	Lasagne	0.34 (0.045)	0.49 (0.003)	0.41 (0.015)	0.41 (0.014)	−0.11 (0.537)	0.26 (0.125)	0.76 (<0.001)	0.01 (0.962)	−0.06 (0.722)
	Macaroni cheese	0.45 (0.039)	0.63 (0.002)	0.57 (0.007)	0.57 (0.007)	0.19 (0.400)	0.24 (0.285)	0.70 (<0.001)	0.22 (0.330)	0.07 (0.766)
	Sweet and sour chicken	0.53 (0.012)	0.61 (0.003)	0.43 (0.048)	0.39 (0.072)	0.27 (0.227)	0.26 (0.250)	0.67 (<0.001)	−0.04 (0.862)	0.09 (0.705)
All meals	–	0.36 (<0.001)	0.38 (<0.001)	0.31 (<0.001)	0.25 (0.001)	0.02 (0.820)	0.15 (0.058)	0.64 (<0.001)	0.23 (0.004)	0.14 (0.067)
